# Application of Ultrasonographic Evaluation in Conservative Treatment for Femoral Fracture During Cesarean Section: A Case Report

**DOI:** 10.1155/cro/4047870

**Published:** 2026-09-28

**Authors:** Yusuke Sakamoto, Takuya Uematsu, Takahiko Yamada, Makoto Hirao

**Affiliations:** ^1^ Department of Orthopedic Surgery, Nippon Medical School, Bunkyo, Tokyo, Japan, nms.ac.jp

## Abstract

The incidence of neonatal femoral fractures during delivery is extremely rare, and cesarean section further decreases this risk. These fractures usually have favorable prognoses due to strong callus formation and natural remodeling. However, no standardized treatment protocol exists. Conservative management with the Pavlik harness has yielded positive results. Although plain radiography is typically used to monitor healing, neonates are susceptible to radiation exposure. Ultrasonography has been suggested as a radiation‐free alternative for evaluating callus formation. This study presents a case of a neonatal femoral fracture treated conservatively with the Pavlik harness, monitored with plain radiography and ultrasonography. In this case, ultrasonography was able to visualize callus formation and deformity correction in a manner similar to radiography. This indicates the potential for ultrasonography to contribute to reduce the need for repeated radiographs and may thereby reduce radiation exposure in neonates.

## 1. Introduction

Neonatal birth‐related fractures are uncommon, occurring in less than 1% of all deliveries, with femoral fractures being particularly rare [1]. Birth‐related fractures have been linked to challenging deliveries, such as breech presentation or macrosomia, in vaginal births [1]. During vaginal delivery, the torque force applied to the fetal lower limb entrapped within the maternal pelvis is thought to cause proximal spiral fractures of the femur in all cases [1]. C‐section is believed to lower this risk to almost zero [1].

Although neonatal birth femoral fractures are uncommon, they generally have a favorable prognosis owing to strong callus formation and the body′s natural healing process [2]. Different treatment approaches, including bed rest, traction, hip spica cast, splint fixation, external fixation, and the Pavlik harness, have been documented; however, each treatment option has its own advantages and disadvantages [3]. The effectiveness of conservative treatment with the Pavlik harness was reported by Stannard et al., with advantages including treatment without anesthesia, shortened hospital stay, and the ability to adjust the harness based on displacement. Furthermore, it does not disrupt maternal–infant bonding, facilitates easy diaper changes at home after discharge, and decreases skin complications [4]. Nonetheless, long‐term monitoring is essential given the potential risk of femoral head necrosis [5].

Plain radiography is commonly used to assess treatment progress. However, very young children are at additional risk because they are inherently more radiosensitive [6]. In recent years, ultrasonography has gained recognition as a valuable tool for evaluating fracture healing [7]. To enable reliable follow‐up using ultrasonography, we examined the concordance between radiography and ultrasonography and assessed the feasibility of ultrasonography in reflecting radiography. By demonstrating the high concordance between these two modalities in a rare case of femoral fracture during C‐section, this report is aimed at providing clinical evidence that ultrasonography can be a safer, radiation‐free alternative for monitoring neonatal bone healing.

## 2. Case Presentation

A female neonate born at 37 weeks of gestation, weighing 2502 g, had a breech presentation as the sole perinatal complication, which necessitated an emergency C‐section owing to premature membrane rupture. The Apgar scores were 9 at 1 min (with a 1‐point deduction for appearance) and 10 at 5 min. Postnatal tests showed normal bone metabolism. The obstetrician noted that the left leg was delivered first, whereas the right leg became entrapped and required forceful extraction. On postnatal Day 2, redness, swelling, and decreased movement of the right lower limb prompted plain radiographs, revealing a fracture at the proximal third of the right femoral shaft with 87° anterior angulation (Figure 1). The patient was transferred to our department the same day. On postnatal Day 4, conservative treatment with a Pavlik harness was initiated. A follow‐up radiograph showed the fracture deformity decreasing to 15° of anterior angulation. From postnatal Day 7 onward, we conducted regular follow‐ups using radiography and ultrasonography to monitor healing. The detailed timeline of clinical events and comparative imaging findings throughout the treatment course is summarized in Table 1. All ultrasonographic examinations were consistently performed by a single operator, a second‐year orthopedic surgery resident (postgraduate year 4). Ultrasonography was performed using a Venue 50 ultrasound system (GE Healthcare, United States) with a linear‐array transducer operating at 13.0 MHz. Ultrasonographic evaluation of the fracture site was performed in three planes: longitudinal view of the anterior thigh and longitudinal and transverse views of the lateral thigh (Figure 2). On postnatal Day 7, when ultrasonography was initiated, no callus formation was observed on either plain radiography or ultrasonography (Figures 3a–d). On postnatal Day 12, early callus formation was first detected on plain radiography and ultrasonography, appearing as a heterogeneous hyperechoic area on ultrasonography (Figures 3e–h). By postnatal Day 15, pronounced callus formation was observed (Figures 3i–l), and the patient was discharged home on postnatal Day 16. Subsequent radiographic and ultrasonographic examinations demonstrated progressive callus growth and gradual remodeling of the fracture site (Figures 4a–d). From the fifth week postinjury, postconfirmation of sufficient bridging callus, nighttime‐only use of the harness was initiated (Figures 4e–h). Bone union was considered to have been achieved on Day 46, when the previously coarse and heterogeneous hyperechoic area became a smooth, continuous cortical line, and the use of the harness was discontinued (Figures 5a–d). Even after the harness was discontinued, gradual remodeling of the fracture alignment was observed (Figures 5e–h). At 7 months of age, physical examination revealed no evidence of motor impairment, limb length discrepancy, or rotational deformity (Figure 6). The patient will be carefully monitored with radiographic examinations at approximately 6‐month intervals for at least 2 years to evaluate for avascular necrosis (AVN) of the femoral head [5].

**Figure 1:**
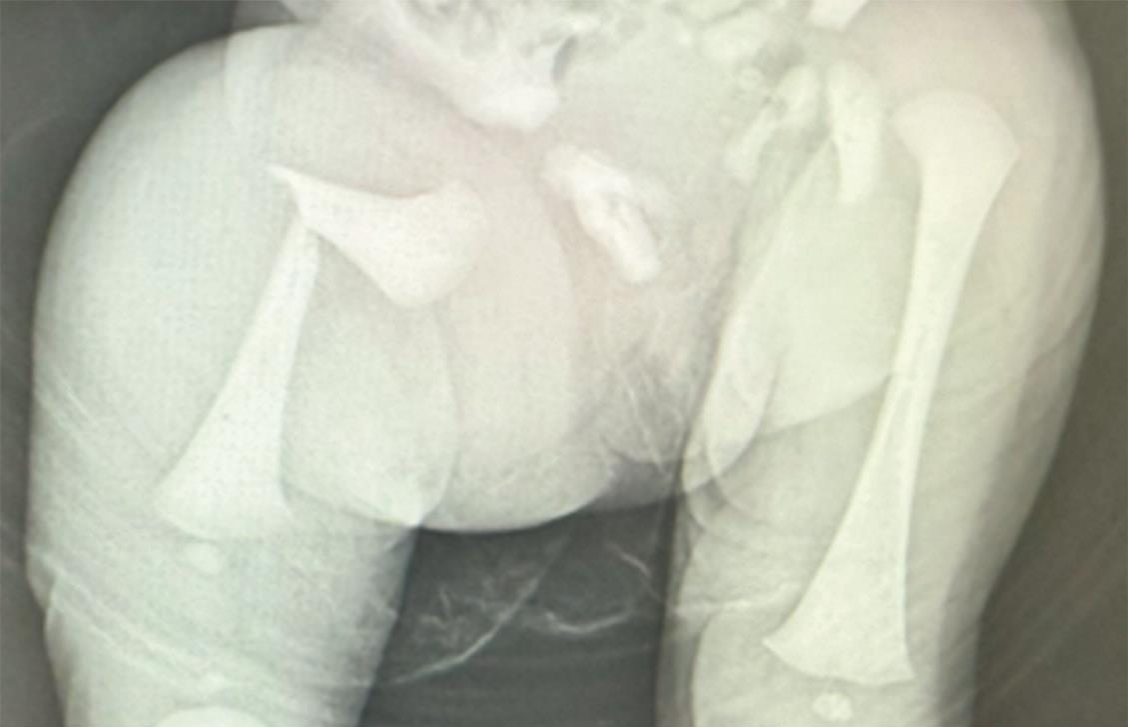
A plain radiograph on postnatal Day 2 showed right femoral fracture.

**Table 1:** Timeline of clinical events and comparison of radiographic and ultrasonographic findings.

Time point	Clinical events and interventions	Radiographic findings	Ultrasonographic findings
Day 0	Delivered via emergency C‐section due to premature membrane rupture	Not performed	Not performed
Day 2	Redness, swelling, and decreased movement in the right leg. Referred to orthopedics	Fracture at the proximal third of the right femoral shaft with 87° anterior angulation	Not performed
Day 4	Pavlik harness treatment initiated	Fracture deformity decreased to 15° of anterior angulation	Not performed
Day 7	Regular monitoring using both radiography and ultrasonography initiated		
Day 12		Initial callus formation detected	Heterogeneous hyperechoic area indicating callus formation
Day 15‐16	Patient discharged home (Day 16)	Pronounced callus formation (Day 15)	
Day 35	Shifted to nighttime‐only harness use	Bridging callus confirmed	
Day 46	Harness treatment discontinued	Bone union achieved	Smooth, continuous cortical line replacing the hyperechoic area, indicating bone union
7 months	No motor impairment	No obvious deformity; progressive remodeling	Not performed

**Figure 2:**
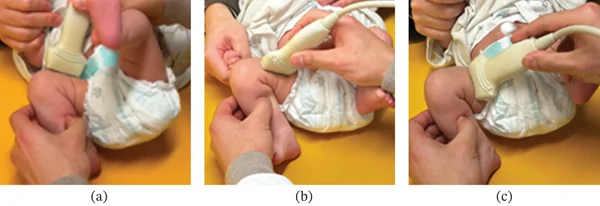
Scenes of the ultrasonography. (a) Longitudinal view of the anterior thigh. (b) Longitudinal view of the lateral thigh. (c) Transverse view of the lateral thigh.

**Figure 3:**
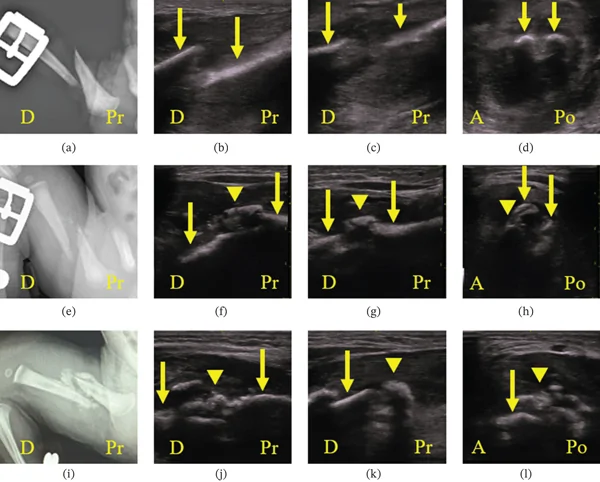
Radiographic and ultrasonographic findings on postnatal Days 7, 12, and 15 Postnatal Day 7: Figures a–d. (a) Plain radiograph. (b) Longitudinal view of the anterior thigh. (c) Longitudinal view of the lateral thigh. (d) Transverse view of the lateral thigh Postnatal Day 12. Figures e–h. (e) Plain radiograph. (f) Longitudinal view of the anterior thigh. (g) Longitudinal view of the lateral thigh. (h) Transverse view of the lateral thigh Postnatal Day 15. Figures i–l. (i) Plain radiograph. (j) Longitudinal view of the anterior thigh. (k) Longitudinal view of the lateral thigh. (l) Transverse view of the lateral thigh. The arrow indicates the cortical bone, and the arrowhead indicates callus. Orientation: Pr, proximal; D, distal; A, anterior; Po, posterior.

**Figure 4:**
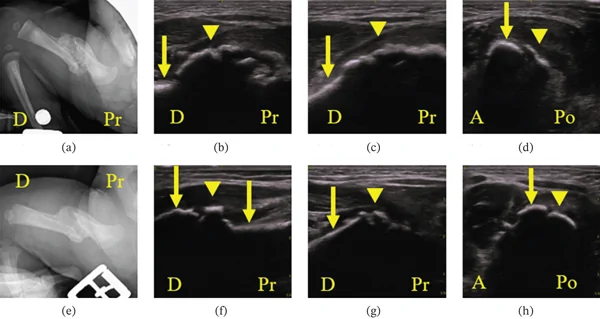
Radiographic and ultrasonographic findings after discharge on postnatal Days 25 and 35, demonstrating progressive callus growth and remodeling Postnatal Day 25: Figures a–d. (a) Plain radiograph. (b). Longitudinal view of the anterior thigh. (c) Longitudinal view of the lateral thigh. (d) Transverse view of the lateral thigh. Postnatal Day 35: Figures e–h. (e) Plain radiograph. (f) Longitudinal view of the anterior thigh. (g) Longitudinal view of the lateral thigh. (h) Transverse view of the lateral thigh. The arrow indicates the cortical bone, and the arrowhead indicates callus. Orientation: Pr, proximal; D, distal; A, anterior; Po, posterior.

**Figure 5:**
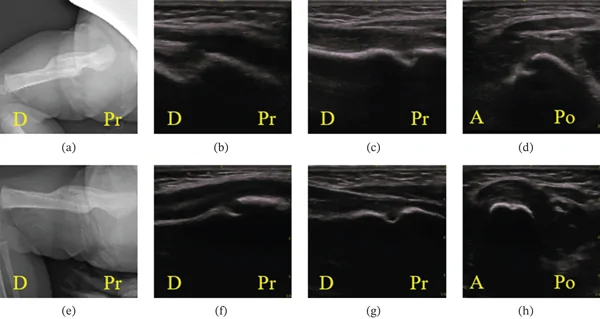
Radiographic and ultrasonographic findings after nighttime‐only use of harness on postnatal Days 46 and 53, demonstrating bone union and subsequent remodeling. Postnatal Day 46: Figures a–d. (a) Plain radiograph. (b) Longitudinal view of the anterior thigh. (c) Longitudinal view of the lateral thigh. (d) Transverse view of the lateral thigh. Postnatal Day 53: Figures e–h. (e) Plain radiograph. (f) Longitudinal view of the anterior thigh. (g) Longitudinal view of the lateral thigh. (h) Transverse view of the lateral thigh. Orientation: Pr, proximal; D, distal; A, anterior; Po, posterior.

**Figure 6:**
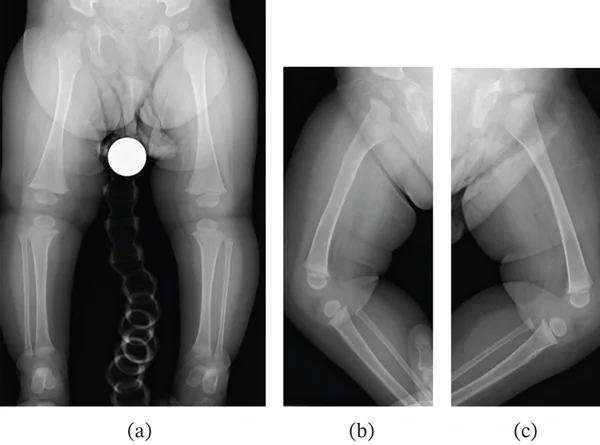
No apparent deformity was observed on plain radiographs at 7 months. (a) Anteroposterior full‐length view of the lower extremities. (b) Lateral view of the right femur. (c) Lateral view of the left femur.

## 3. Discussion

In this study, we confirmed that ultrasonography enabled serial, multiplanar assessment of the fracture‐healing process in delivery‐related femoral fractures, showing good concordance with plain radiography. To our knowledge, no previous report has documented and verified the fracture‐healing course using both ultrasonography and plain radiographs in a delivery‐related femoral fracture; thus, this is the first such report.

Treatment selection for neonatal birth‐related femoral fractures should be strictly individualized depending on multiple factors, including fracture location, angulation, and associated clinical conditions or comorbidities. Kancherla et al. emphasized that treatment selection should be tailored according to the specific fracture location and angulation [8]. Furthermore, in cases complicated by conditions such as myelomeningocele, standard immobilization methods like the Pavlik harness or pelvipedal casting may not be applicable, requiring alternative treatments such as long leg splints to avoid irritation of the lumbar lesion, as reported by Erdem et al. [9]. Therefore, the primary focus of the present manuscript is not to compare or demonstrate the therapeutic superiority of the Pavlik harness over other treatment modalities. Rather, it highlights that utilizing the Pavlik harness enabled serial and multiplanar ultrasonographic evaluation of the fracture healing process—an assessment that would be highly difficult or impractical if a hip spica cast had been used.

Regarding the etiology of this injury, although our orthopedic team could not directly observe the exact intraoperative situation, the mechanism of fracture in this cesarean section likely differs from that of typical vaginal deliveries. Although cesarean delivery is generally expected to reduce the risk of birth‐associated fractures, several reports suggest that limited space for maneuverability, inadequate uterine relaxation, and small incisions can conversely increase the risk during difficult extractions [8]. Although vaginal deliveries typically involve torque forces resulting in spiral fractures, the fracture in our case occurred at the proximal third of the femoral shaft and exhibited anterior angulation. We hypothesize that under such restricted spatial conditions, during the forceful extraction of the entrapped right leg in the breech position, rotational torque was absent. Instead, a bending moment based on the principle of leverage—likely with the maternal pelvis acting as a fulcrum—combined with strong resistance from the neonate′s contracted hip and thigh muscles, contributed to this specific anteriorly angulated fracture at the proximal third of the femur [9]. Although neonatal birth fractures are uncommon, they generally have a favorable prognosis owing to strong callus formation and the body′s natural healing process [2]. Neonates rapidly form callus, so monitoring the fracture status, which changes daily, requires frequent x‐rays. However, lateral plain radiographs can be limited in assessing deformities owing to harness interference, and they primarily focus on callus formation and bone healing. Additionally, neonates are more radiosensitive than adults, raising concerns about radiation exposure. The radiation dose from adult extremity x‐rays is about 0.001 millisieverts (mSv), which is equivalent to less than a single day′s natural background radiation. Furthermore, there is no direct epidemiological data supporting an increased cancer risk at radiation doses below 10 mSv [6]. Nevertheless, radiation exposure in neonates should strictly adhere to the “as low as reasonably achievable” (ALARA) principle [10]. Then, it was considered that if fracture healing was recognized using ultrasound analysis, the problem of radiation exposure should be abolished.

In fracture healing, immature callus permits ultrasound beams to penetrate and visualize the fracture site, which helps assess callus bridging and bone formation union [7]. Furthermore, in a rat model, ultrasound assessment of callus correlated with plain radiographic results, and combining both methods enhanced the evaluation of fracture healing [11]. In this case, ultrasound examination provided views from multiple planes without being obstructed by the harness. It allowed confirmation of the callus formation process and progression of bone union.

There are some limitations in this report. It is a single‐case report and the current follow‐up period is short. Regarding long‐term surveillance for AVN of the femoral head, Endo et al. reported that Legg–Calvé–Perthes disease‐like changes were observed in 18% of hips on anteroposterior pelvic radiographs at 2 years of age, with 7% requiring corrective surgery [5]. Therefore, we plan to carefully monitor the patient with radiographic examinations at approximately 6‐month intervals for at least 2 years.

This study did not directly demonstrate a reduction in radiation exposure. Furthermore, because all ultrasonographic assessments were performed by a single operator, neither interobserver nor intraobserver variability was evaluated. Further study of ultrasound‐only assessment could be sufficient to evaluate the fracture healing in the future with more increased number of cases. However, this study demonstrated the feasibility of substituting ultrasonography for radiography for the assessment of callus formation and bone union. As a next step, we will minimize radiographic follow‐up and transition to an ultrasonographic‐dominant approach. Although ultrasonography corresponded with radiography of callus formation and bone union throughout the course, evaluation of rotational deformity remained challenging with ultrasonography, as with plain radiography. However, recent advances in 3‐D ultrasonography have demonstrated the feasibility of assessing femoral torsion [12], suggesting that 3‐D ultrasonography may enable evaluation of rotational deformity in future studies.

In conclusion, using ultrasonography in combination could potentially reduce the need for plain radiographs, thereby potentially lowering radiation exposure in the conservative treatment of femoral fractures.

## Funding

No funding was received for this manuscript.

## Disclosure

All content was reviewed, verified, and edited by the authors.

## Ethics Statement

This case report was conducted in accordance with the principles of the Declaration of Helsinki. Formal ethical approval was waived by the Institutional Review Board of Nippon Medical School Hospital because this study is a single‐case report and patient anonymity has been strictly maintained.

## Consent

Written informed consent was obtained from the patient′s parents for the publication of this case report and any accompanying clinical and radiological images. A copy of the written consent is available for review by the Editor of this journal.

## Conflicts of Interest

The authors declare no conflicts of interest.

## Patient Perspective

Upon learning of the femoral fracture shortly after birth, the parents understandably experienced significant anxiety. Following a comprehensive discussion regarding the available treatment options, they understood the rationale for conservative management and fully consented to the use of the Pavlik harness. Although initial concerns lingered during the early stages of treatment, the parents reported a growing sense of relief, as they were able to confirm progressive callus formation. They found the addition of noninvasive ultrasonography particularly reassuring; although it was performed alongside standard radiographic monitoring, it provided a detailed, accessible visual evaluation of the healing process. As the fracture alignment gradually remodeled and corrected over time, their fears regarding potential long‐term sequelae subsided. Ultimately, the parents expressed deep satisfaction and immense relief seeing their child grow with normal motor development and no apparent physical limitations.

## Data Availability

The data that support the findings of this study are available from the corresponding author upon reasonable request. The data are not publicly available due to privacy or ethical restrictions.
